# Surgical Treatment in Patients with Toxic Phosphorus Osteonecrosis of Facial Skull Middle Zone

**DOI:** 10.3390/dj11050108

**Published:** 2023-04-23

**Authors:** Davit H. Ispiryan, Gagik Hakobyan, Anastasia Li, Ekaterina Yu. Diachkova, Yuriy Vasil’ev, Artur Kheygetyan, Elena Ivanova, Kirill Zhandarov, Natalia Kireeva, Roman Safronov, Alexey Serikov, Yuri A. Medvedev

**Affiliations:** 1 Department of Maxillofacial Surgery, I.M. Sechenov First Moscow State Medical University (Sechenov University), 121059 Moscow, Russia; 2 Department of Oral and Maxillofacial Surgery, Yerevan State Medical University, Yerevan 0025, Armenia; 3 Department of Oral Surgery, I.M. Sechenov First Moscow State Medical University (Sechenov University), 121059 Moscow, Russia; 4 Department of Operative Surgery and Topographic Anatomy, I.M. Sechenov First Moscow State Medical University (Sechenov University), 119435 Moscow, Russia; 5 Dentistry Department No.1, Rostov State Medical University, 344022 Rostov-on-Don, Russia; 6 Department of Oral Surgery and Implantology, Vladimirsky Moscow Regional Research Clinical Institute, 129110 Moscow, Russia; 7 Department of Therapeutic Dentistry, FGBOU DPO RMANPO of the Ministry of Health of Russia, 125993 Moscow, Russia; 8 Department of Outpatient Therapy, I.M. Sechenov First Moscow State Medical University (Sechenov University), 119333 Moscow, Russia; 9 Department of Physical Education, I.M. Sechenov First Moscow State Medical University (Sechenov University), 119121 Moscow, Russia; 10 Department of Maxillofacial Surgery and Traumatology, A.I. Yevdokimov Moscow State University of Medicine and Dentistry, 127473 Moscow, Russia

**Keywords:** osteonecrosis of the jaws, maxilla, zygoma, orbit, substance related disorder, margins of excision, krokodil, desomorphine

## Abstract

During the last few years, in the territory of the Russian Federation, the number of cases of toxic phosphoric osteonecrosis of the jaws has increased against the background of taking drugs of “artisanal” production (pervitin, desomorphin). The aim of our study was to increase the effectiveness of surgical treatment of patients with a diagnosis of toxic phosphorus necrosis of the maxilla. We performed a comprehensive treatment of patients with a history of drug addiction and the above diagnosis. Surgical intervention in the volume of complete resection of pathologically altered tissues and reconstructive techniques using local tissues and a replaced flap made it possible to achieve good aesthetic and functional results in the early and late postoperative period. Thus, our proposed method of surgical treatment can be used in similar clinical situations.

## 1. Introduction

In recent years, the problem of treating patients with toxic phosphorus osteonecrosis of the bones of the facial part of the skull has become not only medical and highly specific, but also social [1,2,3,4]. This is caused by the occurrence of a pathological process in already immunocompromised patients that could aggravate the course of both the predominant diseases (such as hepatitis C) and the osteonecrosis itself [5].

When analyzing the literature on this issue, we have encountered the absence of such a problem or lack of information in developed countries, where other types of necrosis of the bones of the facial part of the skull associated with the use of bisphosphonates in patients with osteoporosis and distant metastases in malignant tumors, were described [6,7,8]. These categories of patients differ from each other both in age and social status, as well as in the presence of concomitant conditions; however, they have a common feature—a pathological process that has developed in the area of the bones of the facial part of the skull in all patients due to the intake of phosphorus-containing substances [9,10].

Thus, we have found reports on cases of toxic phosphorus osteonecrosis associated with narcotic drugs pervitin and desomorphine injections, occurring only in the Russian Federation and post-Soviet states, that indicates a high level of narcotics abuse in these countries [11,12,13,14,15,16,17]. However, in last 10 years, separate reports (usually clinical cases) have been found in the literature that were connected with artisanal narcotic drugs [18] (and other narcotic drug abuse such as methamphetamine) [19], and the development of a condition is known “meth mouth”, connected to the spreading of cheap drugs in the USA and some regions of Europe [20]. For example, Oliver and co-authors noted the name of “krokodil” drug as the “drug that eats junkies” and “Russia’s Designer drug”, which widely spread in Russia before 2012 before decreasing because of Codeine law regulation changes, but suddenly then appeared in Mexica, the USA, and Germany [21].

Diagnostics and surgical treatment of this category of patients are complicated due to their low compliance, the presence of concomitant infectious diseases (HIV, hepatitis B and C), poor personal and oral hygiene, and severe drug addiction [22]. Generally, such patients do not seek treatment themselves and are brought by relatives, often at an already severe stage of the disease with extensive necrosis, possibly even at the peak of the exacerbation. The usual complaints of such patients are deterioration of their general condition, extensive exposure of the bones of the jaws [23], a large number of fistulas with copious purulent secretion, and an altered configuration of the face due to the development of defects and deformities.

The aim of our study was to increase the effectiveness of surgical treatment of patients diagnosed with toxic phosphorus osteonecrosis of the bones of the facial part of the skull.

## 2. Materials and Methods

The research was performed at I.M. Sechenov University from 2013 to 2016 with the examination and treatment of 50 patients with toxic phosphorus craniofacial osteonecrosis.

The age of patients ranged from 22 to 44 years, meaning that the condition mainly occurred in socially active mostly males of working age (the sex ratio was 41:9, i.e., 4.56:1)

Inclusion criteria:

- aged from 18 years (due to the age of admitted patients);
- history data—an intake of phosphoric narcotics such as desomorphine and pervitin for more than a year;
- medical history—written approval from narcologist at the place of residence—drug use remission for at least 3 months prior to the planned hospital admission;
- the presence of bone tissue disruption according to the patient’s complaints for more than 2 months.

Exclusion criteria:

- lack of compliance;
- constant narcotics abuse and inability/unwillingness to stop using them;
- comorbidity in the decompensation stage or its exacerbation;
- drug addiction relapse;
- voluntary refusal from treatment;
- history of bisphosphonates/denosumab.

The primary diagnostic criteria were the results of clinical examination, medical history, and patient’s complaints: maxilla or craniofacial bone exposure for at least 2 months since the triggering factor, e.g., tooth removal; a presence of intra- and extraoral fistulas with suppuration; foci of infiltration without significant fluctuation. The provisional preoperative diagnosis was “toxic phosphorus craniofacial (according to the affected area) osteonecrosis”. The necessary condition for the diagnosis was pervitin and desomorphine use in history.

**Figure 1:**
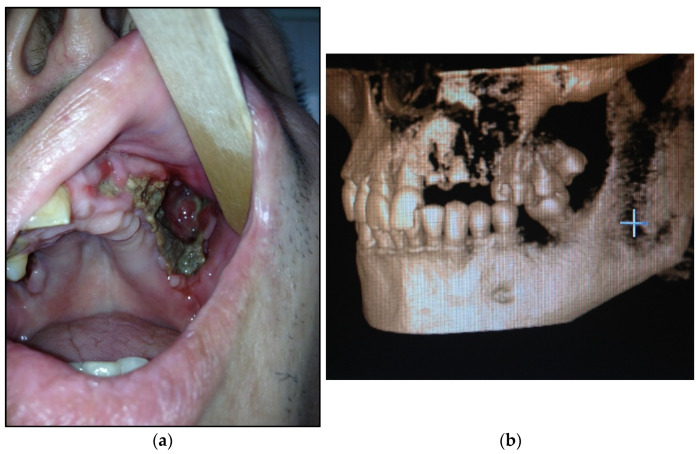
(**a**) Patient with type 1 toxic phosphorus osteonecrosis. Intraoral view; (**b**) CT-scan of patient’s skull.

**Figure 2:**
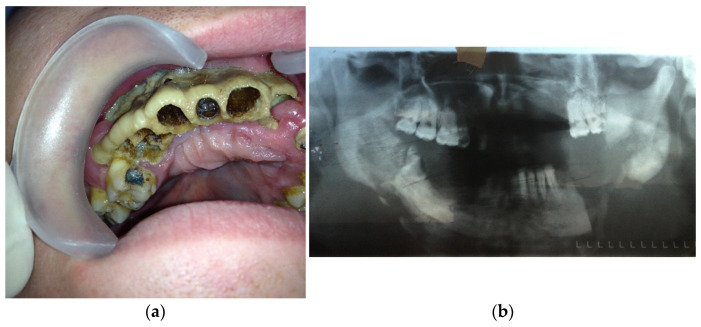
(**a**) Patient with type 2 toxic phosphorus osteonecrosis. Intraoral view; (**b**) patient OPG.

**Figure 3:**
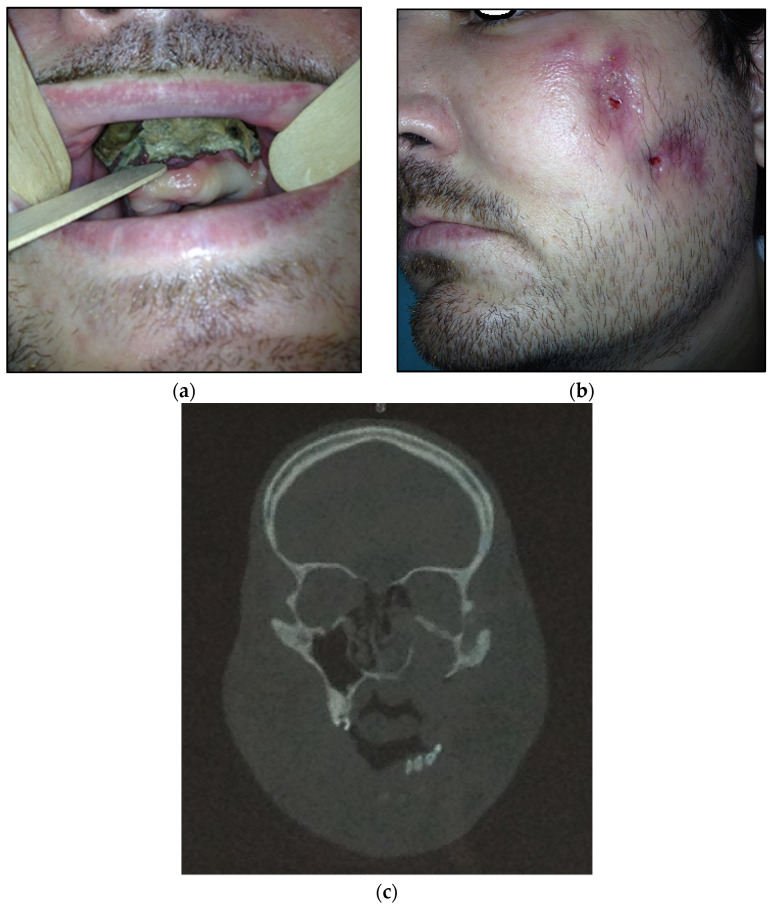
(**a**) Patient with type 3 toxic phosphorus osteonecrosis. Intraoral view; (**b**) extraoral view, (**c**) multi CT-scan of patient’s skull before operation.

**Figure 4:**
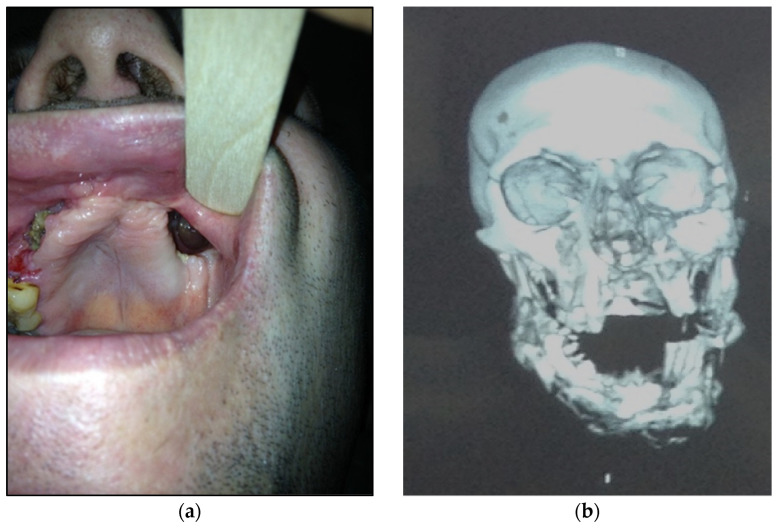
(**a**) Patient with type 4 toxic phosphorus osteonecrosis. Intraoral view; (**b**) multi CT-scan of patient’s skull before operation.

All of the patients were divided into 4 groups based on the type of the pathological process in the midface area:

- Type 1: patients with local pathological process in maxilla (1 or 2 teeth/dental sockets are included)—15 patients (30%) (Figure 1a,b);

- Type 2: patients with maxilla involved (at least three teeth are included)—14 patients (28%) (Figure 2a,b);

- Type 3: patients with maxilla and partially zygomatic bone (its body) involved—11 patients (22%) (Figure 3a–c);

- Type 4: patients with maxilla, zygomatic bone, floor of the orbit involved—10 patients (20%) (Figure 4a,b)

In the preoperative period and during the hospitalization, the patients underwent a standard investigation for non-biased assessment and monitoring. Urinalysis, standard hematology, and biochemistry blood tests were performed for the laboratory diagnosis on the first day of hospitalization and were repeated every 10 days in the facility.

For all patients in this study, hospitalization due to the diagnosis of “toxic phosphorus osteonecrosis of facial skull middle zone’ was the first in their history.

Pre-surgical preparation included all procedures before hospitalization (professional hygiene and antiseptic oral cavity washing 3–4 times per day for 1 week).

Microbiological tests (standard bacteriological quantitate and quality investigation) were also performed before and after hospitalization.

The data were obtained through clinical examination (process localization and symptoms) and investigations (demarcation line—exact border of the pathological process that could be visualized according to CT-scan as the beginning of osteosclerosis or osteolysis) was taken into consideration while planning treatment for the patients in this category.

Due to the subjective nature of the pain syndrome assessment, we applied the widely used numerical rating scale (NRS, numerical rating scale, NRS), which is also designed to determine the intensity of pain and consists of 11 points from 0 “no pain” to 10 “pain that cannot be tolerated” [24]. The swelling in the early postoperative period was marked by relative points as 0—“absence”, 1—“mild”, 2—“significant” (the comparison was with the condition of soft tissue before operation and could be only additional measurement as it was swelling because the pathological process even before operation).

The follow-up was once every 6 months.

Every patient underwent radiologic investigation (skull radiography, panoramic radiography of the jaws, computed tomography of the middle-third of the facial skeleton) to ascertain the location of the pathological process in the middle third of the facial skeleton and to determine the surgical volume. All of the patients have provided lateral and anterior–posterior skull radiographs made in clinics at the place of residence for the initial consultation.

In order to assess the surgical treatment results, and possible progression or remission of the condition, an X-ray was conducted immediately after surgery, if the patient’s state stabilized, in 1, 3, and 6 months, and once in 3 months in case of long-lasting remission—112 radiographs and 42 CT scans were analyzed in total.

Statistical analyses were conducted with R program (R version 4.2.2 (31 October 2022) ucrt)—“Innocent and Trusting” Copyright (C) 2022 The R Foundation for Statistical Computing Platform: x86_64-w64-mingw32/x64 (64-bit)). Means, medians, and standard deviation were calculated and the studied rates were displayed in numerical (absolute) values and as a percentage. When comparing the results of the study, 95% was taken as the level of statistical significance with an error level of α = 5% (or 0.05), and the presence of statistically significant differences between one or another expression of a trait in subgroups was considered significant at *p* < 0.05. Comparative analysis of qualitative variables was carried out after normality of distribution checking with the Shapiro–Wilk test using the Chi-square test χ2 and two-tailed Fisher’s test when comparing several groups and their abnormal distribution—the Kruskal–Wallis test. All statistical methods were based on the principles of evidence-based medicine.

## 3. Results

Type 1 patients presented with intraoral purulent secretion with odor (10 patients—66.7%) and intraoral bone exposure (15 subjects—100%). In type 2 patients, pus in the oral cavity also occurred (13 patients—92.8%), as well as exposed bone areas in the maxilla, a severely painful condition, and teeth position derangement (10 subjects—71%). Type 3 patients’ prevalent complaint was maxilla bone exposure (11 patients—100%), and the same subjects complained about purulent drainage in the oral cavity (11 patients—100%), midface soft tissue swelling, face deformation, painful disorder and malocclusion (8 subjects—73%); in type 4 patients almost all of the listed complaints were present (10 patients—100%). The more severe the osteonecrosis was, the higher number of complaints were identified and the more diverse they were. i.e., even no cooperative patients with low compliance cannot be dismissive of themselves and this condition.

All of the indicated symptoms occurred in all four types of involvement in patients with toxic phosphorus osteonecrosis and could, in general, be clinical features for diagnosing osteonecrosis of the middle-third of the facial skeleton.

The results of analysis of pain syndrome in patients of all of the 4 types are presented in Table 1.

Summing up, the intensity of the pain syndrome escalated in patients according to the type of their condition.

An analysis of the pain syndrome was also conducted in patients during the postoperative period, and the results were compared with those before the surgery.

### 3.1. The Results of the Radiological Investigation

The radiological investigation, including the results of multi-scan computed tomography, showed changes of the bone of maxilla, zygomatic bone, and also, involvement of the orbit in severe type 4 osteonecrosis, with different cortical bone damage similar to chronic osteomyelitis. The radiological features of the osteonecrosis according to the pathological process type are shown in Table 2).

### 3.2. Surgical Approaches in Patients with Maxillofacial Osteonecrosis

The surgical treatment volume varied according to the results of complex clinical examination, laboratory investigations and imaging, location, and spread of pathological process, and the presence of comorbidities, including purulent inflammation in the maxillofacial or any other area.

The surgical treatment volume depended on the type of pathological process in the maxillofacial area.

Group 1 consisted of patients who underwent partial resection of the upper jaw, usually unilateral, from intraoral access (15 patients).

Group 2 was formed by patients who underwent resection of the upper jaw to the border with the zygomatic bone in the projection of the lower third of the maxillary sinus from the extraoral Kocher–Weber access (14 patients).

Group 3 included patients who underwent resection of the upper jaw, part of the zygomatic bone from the extraoral Kocher–Weber approach (11 patients).

Group 4 included patients who underwent resection of the upper jaw, zygomatic bone, and lower wall of the orbit (10 patients).

All “dead” bony tissue was removed between 0.5 and 1 cm beyond the visible borders of osteonecrosis, as the possibility of discrepancy between the results of X-ray and microscopic changes was taken into consideration. This approach made it possible to achieve a stable positive effect in the postoperative period, provided that the patient refused to continue taking narcotic drugs and they maintained oral hygiene.

The effectiveness of treatment was assessed according to the results of clinical examination (reduction of swelling or infiltration in the soft tissue of middle-third of the facial skull, cessation suppuration, absence of bone exposure areas), laboratory investigations (a decrease in the inflammatory features in complete blood count, contamination), and radiological investigation.

### 3.3. Early Postoperative Period (before 14 Days after Surgery)

In the early postoperative period, the patients stayed about 2 days in the ER department under monitoring of common conditions in order to reach a stable state after the extensive surgery was performed. The patients were intubated in order to reduce asphyxia risk due to the postoperative edema from several hours to 2 days, depending on the patients’ condition.

Swelling increased in all of the patients in the postoperative period and reached its maximum on day 2 or 3 after the surgery. It decreased within the next 5–7 days; however, the largest degree of swelling was registered in type 3 and 4 patients due to the extensive resection and local temporal flap reconstruction. The least swelling was presented in patients with a focal osteonecrosis in the middle-third of the facial skeleton and partial intraorally performed resection.

As Kocher–Weber approach was made, eyelid swelling was indicated due to the lymph outflow obstruction.

**Figure 5:**
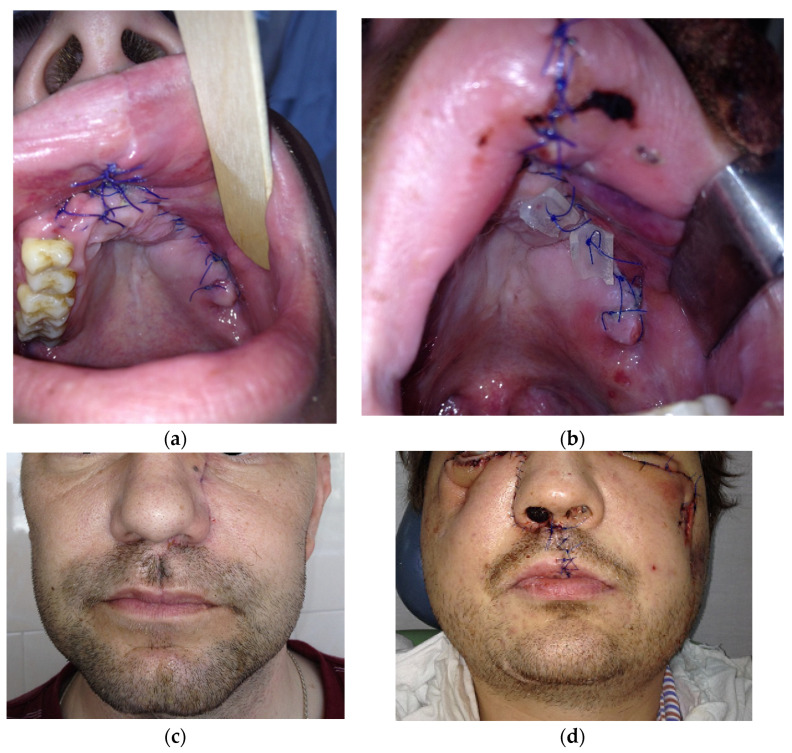
Postoperative view of patients in the early period (before 14 days): (**a**) with type 1; (**b**) with type 2; (**c**) with type 3; (**d**) with type 4.

The view of patients with all four types of pathological process is presented in Figure 5.

The postsurgical pain was also assessed, but in the long-term period, since the results could be affected by the postoperative pain and swelling. The distribution of patients in groups according to the intensity of pain is presented in Table 3 and Table 4.

Despite intensive treatment and measures taken, some patients noted a suture line disruption (6 patients—12%), which we associated with a large volume of surgical intervention, insufficient nutrition for displaced flaps, and, most importantly, non-compliance with basic hygiene rules by patients, i.e., poor compliance.

The majority of patients with sutures failure were in type 3, which could be the result of the reasons mentioned above or an insufficient volume of tissue for defect reconstruction; it was not found in type 4 patients in which local and temporal fascial flap reconstruction was made.

### 3.4. Late Postoperative Period (1 Month–2 Years after Surgery)

The assessment of the late postoperative period was made no sooner than a month after the surgery.

In all of the patients that followed recommendations and did not start using desomorphine again, a steady remission was indicated: according to the results of clinical examination and radiological tests, there was no osteonecrosis spread and the oral health status was comparatively reassuring.

In case of violation of rules and prescriptions, poor oral hygiene, recurrent drug injections, and when these patients were hospitalized repeatedly, saw further spread of osteonecrosis to the other bones of the facial skeleton and even the cranial base. There were also several fatal cases due to a drug overdose (3 patients—6%).

## 4. Discussion

The problems of treating patients with extensive pathological processes of the maxillofacial region requires a surgical intervention and remains relevant throughout the history of medicine. In particular, some scientists have proven the high efficiency of maxillary resection in the case of diagnosed osteonecrosis of the skull bones of various etiologies [3,25,26,27,28,29], which have served as the basis for the development of various surgical techniques for the treatment of patients with toxic phosphorus osteonecrosis of the jaw in Russia and the former USSR countries. However, it is important to note that in addition to the complete removal of affected tissues, it is necessary to promptly and quickly eliminate their deficiency [30], so the issue of facial skeleton reconstruction remains open, including the patients with toxic phosphorus osteonecrosis of the upper jaw after extensive resection methods of treatment [31]. However, spontaneous bone formation was also found in some of the patients who underwent surgical treatment for osteonecrosis [32,33].

While treating patients in our research, we assessed effectiveness according to the results of the clinical examination (reduction of swelling and infiltration of soft tissue in the midface area, cessation of suppuration, absence of maxilla bone exposure areas), laboratory investigations (a decrease in the inflammatory indicators in complete blood count, contamination), radiology tests, and positive outcome was indicated in function (to the extent of a further rehabilitation through the use of conditionally removable prosthesis) and esthetics. It is important to note that in spite of the high sensitivity of MSCT, the prevalence of the pathological process at the micro level can be much greater, which can only be detected intraoperatively and based on the results of medical history examination.

It should be noted that we chose a radical resection to completely eliminate the pathological foci as a surgical method of treatment of patients with toxic phosphorus osteonecrosis of the upper jaw, which coincided with the opinion of some of the authors [34,35]: Malanchuk V.A. and co-author (2014) considered the intraoral resection of the maxilla and other bones in the volume of necrectomy and sequestrectomy as the most effective surgical treatment according to pain syndrome and other symptoms decrease, but they did not accent of late postoperative period and did not control the margins of resection or appearance of new foci.

In our study, type 4 patients were the most severe ones, requiring a large surgical intervention and consequently, an extensive rehabilitation period and a high risk of complications. On the other hand, the type 1 patients were of “light” severity and had higher chances of good outcomes in the postoperative period. It has to be noted that because of the specifics of the patients in this category, such as comorbid chronic infectious diseases, low compliance, and frequent drug addiction relapses, it was troublesome to get the patients to visit the clinic regularly for a follow-up examination—for the first year it was all patients, but with the help of their relatives, however, in the next 2 years, there was a loss of contact for at least the half of patients.

Thus, the issue of the treatment of patients with toxic phosphorus osteonecrosis of the upper jaw is complex and requires a comprehensive treatment: radical resection conduction, reconstructive techniques in order to reduce the period of patients’ rehabilitation, cooperation with narcologists, infectious disease specialists to resolve symptoms of comorbidities, infectious conditions, and drug abuse; counseling for drug addiction overcoming and compliance improvement.

Our study has some limitations: we did not compare the results according to infectious diseases presence (HIV, hepatitis B/C), age, or sex of patients. We had no control group, as according to medical rules, we could not leave patients without appropriate treatment; in these cases, it was sufficient surgical management.

## 5. Conclusions

The performed research showed the importance of surgical treatment in patients with toxic phosphorus osteonecrosis of the facial skull middle zone that was especially effective on the pathological process that spread on a limited area of the jaw. It allowed total removal of the main complaint—the pain—in more than 50% of cases. For severe facial bone damage, the surgical treatment also had satisfactory results as a palliative measure.

## Figures and Tables

**Table 1:** Distribution of patients of different types of osteonecrosis depending on the severity of pain syndrome, according to NRS.

NRS Score	Type 1 (n = 15)	Type 2 (n = 14)	Type 3 (n = 11)	Type 4 (n = 10)	*p*
No pain	5 (33.3%)	4 (28.6%)	2 (18.2%)	0	**<0.05**
Mild pain	2 (13.3%)	2 (14.3%)	1 (9.1%)	0	**<0.05**
Moderate pain	2 (13.3%)	3 (21.4%)	2 (18.2%)	0	**<0.05**
Intense pain	3 (20%)	2 (14.3%)	2 (18.2%)	1 (10%)	>0.05
Very intense pain	2 (13.3%)	2 (14.3%)	2 (18.2%)	1 (10%)	>0.05
Unsufferable pain	1 (6.8%)	1 (7.1%)	2 (18.2%)	8 (80%)	>0.05

**Table 2:** Radiographic signs of damage.

Symptom	Groups				*p*
	1	2	3	4	
Demarcation line in maxilla	15 (100%)	14 (100%)	11 (100%)	10 (100%)	*
Demarcation line in the zygomatic bone	0 (0%)	4 (28.6%)	11 (100%)	10 (100%)	*
Demarcation line in the floor of the orbit	0 (0%)	0 (0%)	0 (0%)	10 (100%)	*
Presence of sequestrum	6 (40%)	4 (28.6%)	5 (45.5%)	8 (80%)	<0.05
Cortical plate sclerosis	15 (100%)	14 (100%)	11 (100%)	10 (100%)	*
Periosteal reaction	15 (100%)	14 (100%)	11 (100%)	10 (100%)	*

* Not available.

**Table 3:** Distribution of patients of different types of osteonecrosis depending on the severity of pain syndrome in the early postoperative period, according to NRS.

NRS Score	Type 1 (n = 15)	Type 2 (n = 14)	Type 3 (n = 11)	Type 4 (n = 10)	*p*
No pain	5 (33.3%)	4 (28.6%)	2 (18.2%)	0	*
Mild pain	2 (13.3%)	2 (14.3%)	1 (9.1%)	0	*
Moderate pain	2 (13.3%)	3 (21.4%)	2 (18.2%)	0	*
Intense pain	3 (20%)	2 (14.3%)	2 (18.2%)	1 (10%)	**<0.05**
Very intense pain	2 (13.3%)	2 (14.3%)	2 (18.2%)	1 (10%)	**<0.05**
Unsufferable pain	1 (6.8%)	1 (7.1%)	2 (18.2%)	8 (80%)	**<0.05**

* Not available.

**Table 4:** Distribution of patients of different types of osteonecrosis depending on the severity of pain syndrome in the late postoperative period, according to NRS.

NRS Score	Type 1 (n = 15)	Type 2 (n = 14)	Type 3 (n = 11)	Type 4 (n = 10)	*p*
No pain	10 (66.7%)	7 (28.6%)	5 (45.4%)	1 (10%)	**<0.05**
Mild pain	3 (20%)	3 (21.4%)	3 (27.3%)	3 (30%)	**<0.05**
Moderate pain	2 (13.3%)	3 (21.4%)	1 (9.1%)	5 (50%)	**<0.05**
Intense pain	0 (0%)	1 (7.1%)	2 (18.2%)	1 (10%)	*
Very intense pain	0 (0%)	0 (0%)	0 (0%)	0 (0%)	*
Unsufferable pain	0 (0%)	0 (0%)	0 (0%)	0 (0%)	*

* Not available.

## Data Availability

Not applicable.

## Abbreviations

NRS: Numeric rating scale for pain.
